# Application of Wireless Dynamic Sleep Monitor in Acupuncture Treatment of Insomnia after Ischemic Stroke: A Retrospective Study

**DOI:** 10.1155/2021/5524622

**Published:** 2021-04-01

**Authors:** Yujuan Song, Xuebing Wang, Friedrich Schubert

**Affiliations:** ^1^TCM Department, Shenzhen Longhua District Central Hospital, Guanlan Avenue No. 187, Shenzhen, Guangdong, China; ^2^Rehabilitation Department, Shenzhen Longhua District Central Hospital, Guanlan Avenue No. 187, Shenzhen, Guangdong, China; ^3^School of Medicine, Dresden Technical University, Fiedlerstraße 27, 01307, Dresden, Germany

## Abstract

**Purpose:**

Retrospective analysis of the clinical effect of acupuncture on insomnia after ischemic stroke by using a wireless sleep monitor as an innovative evaluation method.

**Methods:**

From March 1, 2018, to September 30, 2019, 105 cases of insomnia after ischemic stroke were extracted from the inpatient medical record system of Shenzhen Longhua District Central Hospital. According to differences in the treatment plan, the cases were divided into an acupuncture group (57 cases) and a drug group (48 cases). The acupuncture group was given acupuncture treatment on the basis of usual care, while the drug group was given estazolam oral treatment on the basis of usual care. Under the ICF framework, the related items of sleep function and emotion function were selected for evaluation. As outcome parameters, the alterations of the Pittsburgh sleep quality index (PSQI), the self-rating anxiety scale (SAS), and the self-rating depression scale (SDS) were used before the treatment, after treatment, and in a follow-up; meanwhile, the ActiSleep-BT wireless sleep monitor was used to measure total sleep time (TST), sleep efficiency (SE), and sleep arousal (SA) of the two groups before and after treatment and at follow-up.

**Results:**

Within-group comparison showed significant differences in the acupuncture group before treatment and after treatment on the ActiSleep-BT wireless dynamic sleep monitor data as well as in PSQI and ICF. Comparing the acupuncture group with the control group also showed significant differences in the ActiSleep-BT wireless dynamic sleep monitor data, PSQI, and ICF.

**Conclusion:**

By evaluation using ActiSleep-BT wireless sleep monitor, acupuncture treated insomnia after ischemic stroke; the effect is better than usual care.

## 1. Introduction

Insomnia is one of the common diseases after stroke, which is characterized by difficulty in falling asleep, difficulty in sleeping, and difficulty in recovering energy through sleep. 50%–68% of stroke patients are accompanied by insomnia and sleep dysfunction to varying degrees, accounting for about 10% of the total number of patients with chronic insomnia [1]. Insomnia can not only affect patients' physical and mental health, quality of life, and nerve function, but also aggravate the risk of a recurrence of a cerebral infarction or cerebral hemorrhage [2]. Most drugs used in clinical treatment of insomnia cause dependence and tolerance. Long-term use can lead to drug resistance or addiction, and the recurrence rate is high after stopping medication [3–5]. Therefore, considering the defects of insomnia drugs and various adverse reactions, non-drug treatment has gradually become the key treatment for insomnia after stroke [6, 7]. Our group retrospectively analyzed the medical records of acupuncture treatment of insomnia after ischemic stroke, in order to further evaluate its clinical efficacy.

## 2. Clinical Data

### 2.1. Research Object and Grouping

Patients with insomnia after ischemic stroke who were hospitalized in the Rehabilitation Department, Traditional Chinese Medicine Department, or Neurology Department of the Shenzhen Longhua District Central Hospital from March 1, 2018, to September 30, 2019, were extracted from the inpatient medical record system of Shenzhen Longhua District Central Hospital. Finally, according to the inclusion and exclusion criteria, a total of 105 subjects were determined, including 57 cases in the acupuncture group and 48 cases in the drug group.

### 2.2. Diagnostic Criteria

#### 2.2.1. Diagnostic Criteria of Ischemic Stroke

The diagnostic criteria of ischemic stroke were formulated with reference to the Chinese Guidelines for Diagnosis and Treatment of Acute Ischemic Stroke 2014 [8, 9]: (a) acute onset; (b) focal neurological deficit (weakness or numbness of one side of the face or one limb, language disorder, etc.), or comprehensive neurological deficit; (c) unlimited duration of symptoms or signs (when imaging shows responsible ischemic lesions), or duration of symptoms for more than 24 hours (when lack of imaging responsible lesions); (d) elimination of nonvascular causes; (e) brain CT/MRI examination having excluded cerebral hemorrhage.

#### 2.2.2. Diagnostic Criteria for Insomnia

The diagnostic criteria of insomnia are in line with the diagnostic criteria of insomnia proposed in the Chinese Classification and Diagnostic Criteria for Mental Disorders (CCMD-3). The typical symptoms of patients are sleep disorders such as difficulty in falling asleep, dreaminess, insufficient sleep depth, easy waking from sleep, early waking, and difficulty in falling asleep after waking up, accompanied by daytime sleepiness and fatigue. Sleep disorders occur more than 3 times a week and last for more than 30 days. Insomnia induces a series of emotional problems or part of activity efficiency is obviously reduced, or it is accompanied by mental disorder and even hinders the performance of social function.

### 2.3. Inclusion Criteria

The inclusion criteria were as follows: (a) the diagnostic criteria above are met; (b) inclusion into the study within 2 weeks to 6 months of onset; (c) medical records are complete, the acupuncture prescription is entered at least once a week, and there are assessment scales for admission and hospitalization; (d) the length of hospitalization is at least 7 days; (e) participation is voluntary and informed consent has been signed.

### 2.4. Exclusion Criteria

The exclusion criteria were as follows: (a) patients with severe cardiovascular/cerebrovascular diseases, severe liver, kidney, hematopoietic system, or other diseases; (b) patients with mental illness in acute attack; (c) patients who are afraid of acupuncture and do not cooperate with acupuncture treatment; (d) incomplete medical records and lack of important diagnosis and treatment information; (e) the case records cannot meet the Guidance Revised Standards Reporting Interventions Clinical Trials Acupuncture (STRICTA) requirements.

## 3. Treatment

### 3.1. Basic Treatment

Both groups were given basic treatment for primary diseases, such as controlling blood pressure, improving cerebral circulation, resisting platelet aggregation, etc., accompanied by corresponding comprehensive nursing and targeted rehabilitation physiotherapy for limb function, and most patients were given comprehensive nursing for ischemic stroke. Comprehensive nursing should include nursing countermeasures for insomnia, such as creating conditions and environment conducive to sleep, training subjects to develop good sleeping habits, teaching and explaining the correct use of sleeping pills, conducting necessary psychological counseling, and highly cooperating with rehabilitation and nursing of limb functions to relieve insomnia caused by limb factors.

### 3.2. Acupuncture Treatment Group

According to the Guidance Revised Standards Reporting Interventions Clinical Trials Acupuncture (STRICTA), the selection of acupoints, operator, needles, the background and clinical experience of the operator were standardized to ensure the quality control of the acupuncture treatment. The following acupoints were used for the standardized acupuncture treatment: Baihui (DU20), Shenting (DU24), Yintang (Hall of Impression), Anmian bilateral (Peaceful Sleep), Shenmen bilateral (HT7), and Sanyinjiao bilateral (SP6). Patients were lying on their back during acupuncture treatment, and all acupoints were disinfected routinely. Acupuncture was performed with a 0.30 mm × 40 mm Huatuo stainless steel filiform needle. The needles were left for 20 min for each treatment, for 3 treatments per week for 4 weeks, which made for a total of 12 treatments.

### 3.3. Control Group

The control group was treated with benzodiazepine pills (Huazhong Pharmaceutical Co., Ltd., approval number: Sinopharm Zhunzi H42021522, 1 mg) oral, with the initial dose of 1 mg taken 30 minutes before going to bed every night, and the adjusted dose of 1-2 mg every night.

## 4. Outcomes

We set ActiSleep wireless dynamic sleep monitor data as primary outcome; and the secondary outcome was Pittsburgh Sleep Quality Index (PSQI), and the International Classification of Functioning, Disability and Health Rehabilitation Set (ICF-RS).

## 5. Evaluation

### 5.1. ActiSleep Wireless Dynamic Sleep Monitor

The ActiSleep sleep monitor made by Beijing Baianji Technology Co., Ltd., was worn on one arm by patients, and the sleep efficiency (SE), sleep awakening times (SA), and total sleep times (TST) of patients were monitored before, during (2^nd^ week of treatment), and after treatment.

### 5.2. Pittsburgh Sleep Quality Index (PSQI)

Before and after treatment, the sleep quality of the subjects was evaluated from 7 aspects: subjective sleep quality, time to fall asleep, sleep time, sleep efficiency, sleep disorder, hypnotic drugs, and daytime dysfunction [10].

### 5.3. The Patients Life Quality Was Evaluated by the World Health Organization Quality of Life (WHOQOL) Questionnaire

The WHOQOL is used to quantify patient's health-related quality of life. This scale was chosen, because it allows for international comparability, because the achieved scores of health-related quality of life are comparable between patients of different cultural backgrounds [11].

### 5.4. The International Classification of Functioning, Disability, and Health Rehabilitation Set (ICF-RS)

The ICF-RS was developed by the WHO and is based on an international expert investigation and systematic large-scale data analysis: 30 items are selected from more than 1400 categories of the International Classification of Functioning (ICF) to describe the key functions of patients (from acute stage, recovery stage to chronic stage). The quantitative standard evaluation of ICF evaluates rehabilitation combination of adult rehabilitation population according to ICF, including the composition of rehabilitation combination, evaluation rules, judgment of result grade, handling of special circumstances, and precautions for use, etc. [12]. According to the level 1 limit value general scale (grades 0–4) formulated by ICF R&D Center, each item is divided into five grades: no dysfunction (grade 0), mild dysfunction (grade 1), moderate dysfunction (grade 2), severe dysfunction (grade 3), and complete dysfunction (grade 4). At the same time, considering the special circumstances such as the gender and condition of the assessed object, the original grades of 8 (unspecified) and 9 (not applicable) were also used [13, 14], as can be seen in Table 1.

#### 5.4.1. b134 Sleep Function (Category 13)

1. Category definition: the general mental function that produces periodic, reversible, and selective physical and psychological freedom from the environment characterized by physiological changes in which individuals live.
2. Content description: the subject can selectively maintain proper time and quality of sleep to meet daily sleeping needs. This includes the amount, start, maintenance, and quality of sleep and takes into consideration pathologies of sleep, e.g., insomnia, lethargy, and narcolepsy. Excluded are consciousness function (b110), energy and driving force function (b130), attention function (b140), and psychomotor function (b147).
3. Assessment language: have you had any sleep problems in the past 2 weeks?
4. Assessment guidance: in this category, the assessed person needs to comprehensively consider three factors, sleep time, sleep quality, and the troubles caused by sleep problems to life, work, and study, and mark the corresponding numbers 0–10 by using the Numerical Rating Scale (NRS).
5. Scoring rules: Numerical Rating Scale (NRS); please see Table 2.

Level 0: the above NRS score is 0. Level 1: the above NRS score is 1-2. Level 2: the above NRS score is 3–5. Level 3: the above NRS score is 6–9. Level 4: the above NRS score is 10. Level 8: unspecified. Level 9: not applicable.

#### 5.4.2. b152 Emotional Function (Category 14)

1. Category definition: special mental functions related to emotion: components in emotional and psychological activities.
2. Content description: an individual's ability to generate appropriate emotions and manage various emotions. This includes emotional moderation, adjustment and range of emotions (such as sadness, happiness, love, fear, anger, hatred, tension, anxiety, happiness, and sadness), emotional variability, and a functional regulation of emotion, excluding temperament and personality function (B126), as well as energy and drive function (b130).
3. Assessment language: in the past 2 weeks, how would you rate your own ability to generate, control, and regulate emotions?
4. Assessment guidance: the aim is to evaluate whether the assessed individual can produce appropriate emotions and whether there is emotional inversion: can you control and adjust your emotions when you are happy, angry, or sad? Evaluate whether the mood is stable, whether there is temper tantrum, improper speech, disordered expression, physical attack, reticence, etc. The assessed person can be guided to think about the ability to generate, control, and adjust emotions when encountering specific situations or in the current environment (such as a hospital ward). Answers are marked in the following assessment criteria 0 to 10 (NRS).
5. Scoring rules: Numerical Rating Scale (NRS); please see Table 2.

## 6. Statistical Methods

The data were analyzed by SPSS 25.0. The measured data are expressed as mean values and standard deviation (SD). Paired *t*-tests were used for intra-group comparison, and independent sample *t*-tests were used for inter-group comparison. The counting data were expressed by rate or composition ratio, and the comparison between groups was performed using the chi-square test. The comparison between the hierarchical data sets was done using Ridit analysis. Differences were considered as statistically significant if *p* < 0.05.

## 7. Results

### 7.1. Baseline

Baseline data shows the basic situation of all 105 subjects, and the comparison based on grouping. There was no significant difference after statistical analysis, and the two groups are comparable. See Table 3.

### 7.2. Comparison of Dynamic Sleep Monitoring Data

The dynamic sleep monitoring data can be found in Table 4. Three aspects are shown: sleep efficiency (SE), sleep awakening times (SA), and total sleep time (TST). Among the three assessment time points, there was a significant difference between groups. Meanwhile, within both groups, all three aspects showed improvement after treatment compared to before treatment. See Table 4.

### 7.3. Comparison of PSQI and ICF

Both PSQI and ICF data showed the treatment effect of acupuncture group is superior to control treatment. There were significant differences between groups at nearly all compared items. See Table 5.

## 8. Discussion

Insomnia is the most common symptom of stroke patients, and its incidence rate among stroke patients is much higher than in the general population. Insomnia has a great impact on the prognosis of stroke patients [15], as it increases the recurrence rate of stroke, induces psychological disorders and cognitive impairment, aggravates physical symptoms, and seriously affects the rehabilitation process and daily life [16]. The treatment of this late effect of ischemic stroke has not been paid enough attention. Sedative and hypnotic drugs are generally used for symptomatic treatment in clinic [17]. The benzodiazepine Estazolam, which was selected in this study, can block the impulse conduction from the limbic system to the brainstem reticular structure, reduce the conduction excitation in the cerebral cortex, and thereby have sedative and anticonvulsant effects. However, benzodiazepines are known to induce drug dependence and cause various adverse reactions [18].

Modern acupuncture experimental research has proven that the corresponding acupuncture points can increase the content of 5-hydroxytryptamine and aminobutyric acid in the rat brain and reduce the content of glutamate, the excitatory neurotransmitter, so as to improve the central inhibitory function and thus lead to improvement in case of insomnia [19]. According to the meridian syndrome differentiation of traditional Chinese medicine, insomnia is an ailment located in the brain, and the ascending of the governor vessel (Du Meridian) belongs to the brain. Therefore, the Baihui acupoint (Du20) is selected as the main acupoint for this treatment, and the Shenting (Du24) and Yintang acupoints (Hall of Impression) are combined to regulate the governor vessel, regulate the mind, and tranquilize the mind. Anming, as the name implies (Anming means peaceful sleep), has a special therapeutic effect on insomnia, can calm and induce sleep, and is a commonly used acupoint for sleep disorders. The Shenmen acupoint (HT7) is the Yuan (primary) acupoint and acupoint of hand-shaoyin heart meridian, which is mainly used for treating heartache and insomnia [20]. Acupuncture of Shenmen (HT7) can regulate the original qi of heart meridian and tranquilize the mind. Sanyinjiao (SP6) refers to the intersection point of three yin meridians and is effective against various diseases which are caused by yin deficiency, such as palpitation and insomnia [21].

To evaluate the curative effect of the acupuncture treatment, a subjective scale was combined with objective data. As a standard and recognized objective evaluation index of sleep quality, kinescope can effectively ensure the reliability of curative effect evaluation. Previous methods of sleep quality assessment include polysomnography (PSG), or subjective assessment methods such as sleep diary and sleep scale. Although PSG can accurately analyze sleep and waking state, its relatively high price and inability to record sleep in a natural state limit its clinical application. Although the sleep diary can reflect the sleep status in a natural state to a certain extent, its accuracy has not been recognized. However, the kinescope can accurately and continuously record the patient's sleep time and movement during sleep, making it an objective monitoring index that can replace PSG to evaluate sleep quality. As the subjects of this experiment are insomnia patients after stroke, there are many potential influencing factors in the experiment, such as medication baseline of stroke patients, acupuncture experience of patients, etc. [22]. These influencing factors were not controlled in this experiment. However, these factors will be the focus for the improvement of high-quality randomized controlled clinical trials with strict specifications, which are to follow up on this study.

## Figures and Tables

**Table 1:** ICF first qualifier generic scale.

Value	Problem severity	Impact of the problem	Occurrence frequency %
0	No problem	Can be ignored, negligible	0–4
1	Mild	A little	5–24
2	Moderate	Medium	25–49
3	Severe	High, very much	50–95
4	Completely	Totally	96–100
8	Unspecified	Lack of sufficient information	
9	Not applicable	Unsuited	

**Table 2:** Numerical Rating Scale (NRS).

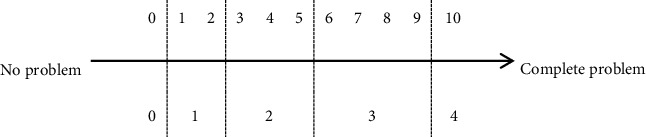

Level 0: the above NRS score is 0. Level 1: the above NRS score is 1-2. Level 2: the above NRS score is 3–5. Level 3: the above NRS score is 6–9. Level 4: the above NRS score is 10. Level 8: unspecified. Level 9: not applicable.

**Table 3:** Baseline of data.

	Acu. group	Ctrl. group	Total
*N*=	57	48	105
Age (mean)	52.06	50.77	51.69
Age (SD)	12.25	16.04	17.22
Male (*n*)	31	27	58
Female (*n*)	26	21	47
Living alone (*n*)	6	3	9
Living with others (*n*)	51	45	96
Still working (*n*)	49	37	86
Disease course (max)	24 w	24 w	24 w
Disease course (min)	2 w	2 w	2 w
Disease course (mean)	13.61w	12.18 w	13.49 w
Disease course (SD)	12.73	9.82	13.50
Hospitalization days (max)	28 d	32 d	29 d
Hospitalization days (min)	7 d	7 d	7 d
Hospitalization days (mean)	26.43	25.71	26.33
Hospitalization days (SD)	5.00	4.92	4.27

**Table 4:** ActiSleep-BT data comparison.

	Acu. group			Ctrl. group		
	Before t.	During t.	After t.	Before t.	During t.	After t.
*N*=	57	57	57	48	48	48
SE% (mean)	77.26	83.62	85.17	78.33	79.07	79.62
SE% (SD)	6.11	7.00	8.61	7.10	6.99	6.57
TST (min) (mean)	351.81	411.63	468.52	363.20	408.73	416.84
TST (min) (SD)	99.30	121.84	109.71	130.64	122.44	127.02
SA (mean)	18.15	17.71	14.20	19.05	16.79	17.83
SA (SD)	10.08	9.37	7.51	11.17	10.86	12.09

**Table 5:** Data comparison of PSQI and ICF.

	Acu. group		Ctrl. group	
	Before t.	After t.	Before t.	After t.
*N*=	57	57	48	48
PSQI (mean)	12.94	10.77	12.05	11.83
PSQI (SD)	4.72	3.08	6.42	5.50
ICF b134 (mean)	2.89	1.04	2.75	1.84
ICF b134 (SD)	1.05	1.31	1.62	1.96
ICF b152 (mean)	2.79	0.68	2.66	1.27
ICF b152 (SD)	1.17	1.29	1.38	1.54

## Data Availability

The study data are available from the corresponding author upon request.
